# Air ambulance and hospital services for critically ill and injured in Greenland, Iceland and the Faroe Islands: how can we improve?

**DOI:** 10.3402/ijch.v74.25697

**Published:** 2015-06-10

**Authors:** Björn Gunnarsson, Niels S Kieler Jensen, Tummas i Garði, Helga Harðardóttir, Lilja Stefánsdóttir, María Heimisdóttir

**Affiliations:** 1 Norwegian Air Ambulance Foundation, Drøbak, Norway; 2 Rigshospitalet, København, Denmark; 3 Landssjúkrahúsið, Tórshavn, Faroe Islands; 4 Landspítali, Reykjavík, Iceland

**Keywords:** health care delivery, emergency medical services, air medical services, Greenland, Faroe Islands, Iceland, Nordic Atlantic Cooperation

## Abstract

The Nordic Atlantic Cooperation (NORA) is an intergovernmental organization under the auspices of the Nordic Council of Ministers. The NORA region comprises Greenland, Iceland, Faroe Islands and western coastal areas of Norway. Historical, cultural and institutional links bind these nations together in multiple ways, and regional co-operation has in recent years become a focus of interest. This commentary addresses air medical services (AMSs) and available advanced hospital services in the 3 smallest NORA countries challenged sparse populations, hereafter referred to as the region. It seems likely that strengthened regional co-operation can help these countries to address common challenges within health care by exchanging know-how and best practices, pooling resources and improving the efficiency of care delivery. The 4 largest hospitals in the region, Dronning Ingrids Hospital in Nuuk (Greenland), Landspítali in Reykjavík and Sjúkrahúsið á Akureyri, (both in Iceland) and Landssjúkrahúsið Tórshavn on the Faroe Islands, have therefore undertaken the project Network for patient transport in the North-West Atlantic (in Danish: Netværk for patienttransport i Vest-Norden). The goal of the project, and of this article, is to exchange information and provide an overview of current AMSs and access to acute hospital care for severely ill or injured patients in the 3 participating countries. Of equal importance is the intention to highlight the need for increased regional co-operation to optimize use of limited resources in the provision of health care services.

Health care organizations aim to develop robust systems to manage patients with a serious illness or injury that poses a threat to life. However, as noted by Lockey, the standard and techniques of emergency care, both within and between countries, is to some extent a geographical lottery (1). Air medical services (AMSs) in Greenland, Iceland and the Faroe Islands are a prerequisite for access to acute secondary and tertiary hospital services for a large proportion of the population in these countries. The number of patient transfers by air ambulance in the region is already substantial and may increase due to the general trend towards specialization and centralization of health care.

Scientific knowledge about pre-hospital emergency care is limited, but studies have shown that, when appropriately used, air medical transport can save lives and reduce costs (2–4). Mortality due to acute coronary heart disease, stroke and many other conditions has declined significantly in recent decades, and part of this reduction can be attributed to better management in the early acute phases of illness or injury (5). Reducing out-of-hospital time for critically ill and injured patients, bringing more medical capabilities to the patient and quick transport to a centre with appropriate services are essential requirements (4,6–9). For seriously ill and injured patients, this will in many cases involve transfer to a tertiary care multi-subspecialty hospital (6,7,9,10). Anaesthetists and neonatologists are commonly involved in transfers of the most critical patients (3,11,12).

All 3 countries face challenges in providing quality health care for a sparsely populated region with limited economic resources. The challenge for governments and health care policy makers is to ensure provision of quality health care for the whole population in an environment that demands greater productivity and fiscal sustainability (13). This pressure for new and better solutions means there is a growing focus on regional co-operation on health (14,15). Geographic and climatic conditions make AMSs in the region a logistical challenge and sometimes a hazardous undertaking (16). Timely communication, co-operation and mutual support between countries is therefore extremely important for optimizing efficiency of transfer and the safety of patients and staff.

The goal of the project, and of this article, is to exchange information and provide an overview of current AMSs and access to acute hospital care for severely ill or injured patients in the 3 participating countries. Of equal importance is the intention to highlight the need for increased regional co-operation to optimize use of limited resources in the provision of health care services.

We elected for 2 reasons not to include the western coastal areas of Norway in this NORA supported project. Firstly, there are already links between the participating hospitals that can potentially be developed. Secondly, the Helicopter Emergency Medical Services (HEMS) in Norway are already well developed, with a median flying time to scene of only 19 minutes (17).

## Methods

The 4 largest hospitals in the region participated in the project: Dronning Ingrids Hospital in Nuuk (Greenland), Landspítali in Reykjavík and Sjúkrahúsið á Akureyri (both in Iceland) and Landssjúkrahúsið Tórshavn on the Faroe Islands. Each partner appointed at least 1 health care worker, with excellent knowledge about organization of AMSs to and from the representative institution, to a working group. All members of the working group are authors of this article. Three of them are specialists in anaesthesia and critical care medicine, with expertise in domestic and international AMSs. Each partner first reviewed the current status of AMSs in their own country and presented a summary of their findings at 3 project meetings, held in Reykjavík, Copenhagen and Tórshavn. The reviews included general information about available airports (e.g. type of runway, distance to regional airports, opening hours, distance from local major settlement), number and type of aircraft, training of staff and legislative issues, organization of hospital services, as well as approximate number and type of patient transports. An AMS quality register, kept at Akureyri Hospital, was used to obtain information about domestic AMSs with fixed-wing aircraft, as well as patient transfers from Greenland to either Iceland or Denmark. Financial services at the hospitals in Reykjavík and Tórshavn provided additional information about number and type of transports and this was supplemented with personal information from group members. The present review was drafted after further exchanges of information by phone and e-mails, with substantial contributions from several co-workers.

## Challenges in the Arctic

**Fig. 1:**
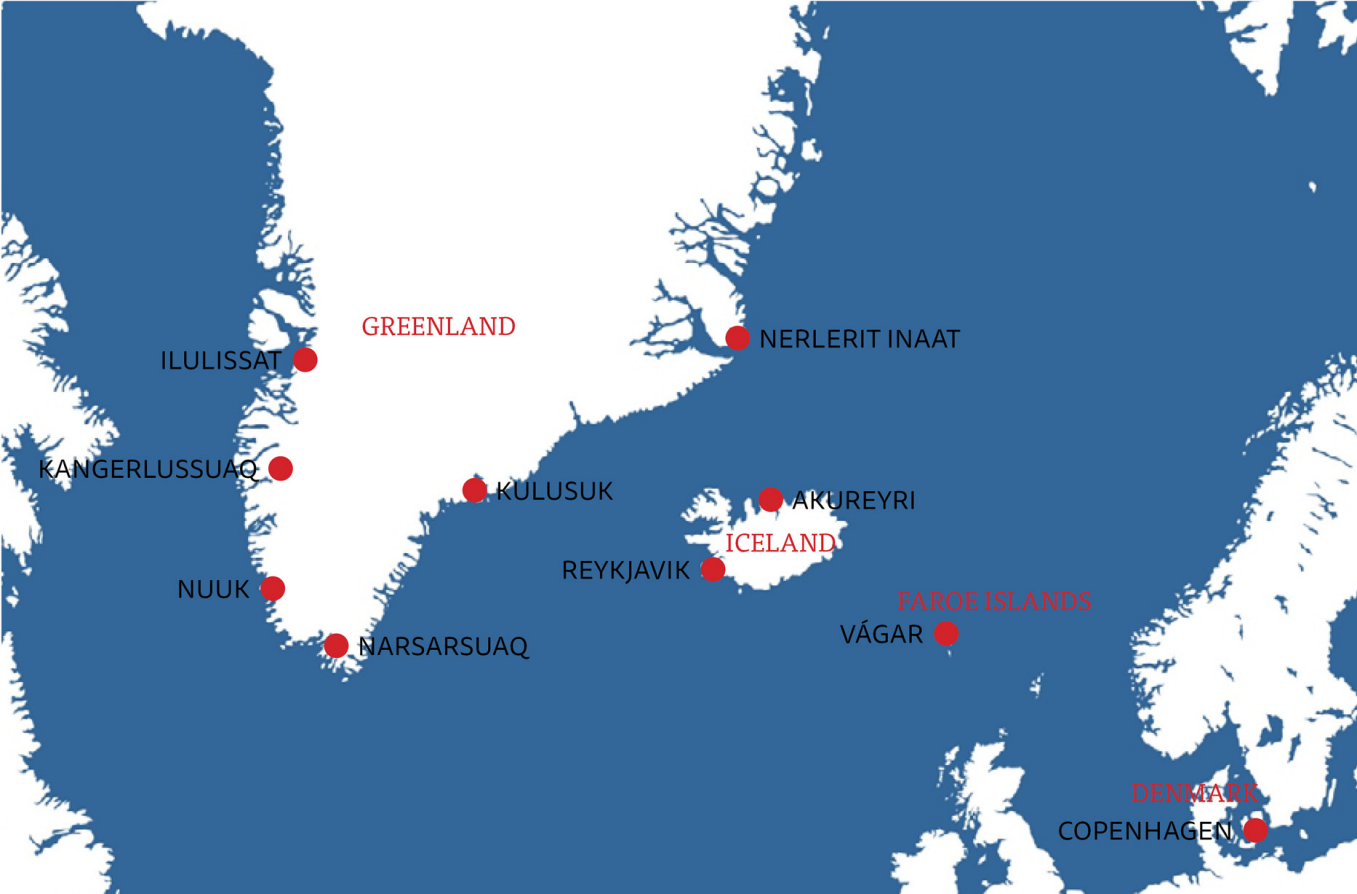
Map of Greenland, Iceland and Faroe Islands showing major airports.

Challenges associated with AMSs in the region include low call volume, remoteness and harsh climate (18). The many weather hazards include visibility-restricting phenomena such as blowing snow, frost, icing and lack of contrast (“whiteout”). In this cold climate, special equipment is required for aircraft and crew and for patient care, and there is a significant risk of icing of aircraft and runways. Transport is often over-sea and the distances involved can be quite long, calling for good flight planning and preparation. Notes that pilots have accumulated over time have at times proved invaluable in this process. All aircraft must have a specified alternate airport in case their planned landing site closes because of adverse weather. Two alternate airports are required in some weather conditions and all 3 airports must be open (Fig. 1). Information about weather and airport runway conditions must come from an air traffic controller, but it can be difficult to get a hold of an air traffic controller after closure of the tower. Some smaller airports depend on daylight and therefore have very short opening hours during the winter. Short runways exclude many types of aircraft from landing, and, for this reason, turboprop airplanes are used for AMSs in Greenland and Iceland. Ambulance jets can however be used at Kangerlussuaq and Narsarsuaq in Greenland, at several airports in Iceland, and at Vágar on the Faroe Islands. Turboprops fly slower than jets, but turbulence, which is not uncommon in Vágar, is better tolerated at slower air speeds.

Medical crews also face multiple challenges. On longer flights, it may be necessary to carry large amounts of oxygen, medical air, medications and other medical supplies, but the aircraft is often small and there are weight restrictions. Communication between health care providers can become difficult at times and experience is limited by low patient volumes. These challenges, among many others, call for good organization to optimize the safety and efficacy of AMSs.

## Target population and available services

### Greenland

Greenland, a part of the Kingdom of Denmark, became self-governing in 2009. It is the largest island in the world, with a total area of 2.2 million km^2^, of which more than 80% is covered by ice. The country's 17 small towns and 60 villages are situated along the ice-free coast, isolated from one another and accessible only by boat or aircraft. The majority of the 56,000 inhabitants live on the south and central west coast. The largest municipality is the capital Nuuk (Godthåb), with 16,000 inhabitants.

Dronning Ingrids Hospital (Dronning Ingridip Napparsimmavissua) in Nuuk, with around 190 beds, is the central hospital for the whole country (Table II). The main tertiary referral hospital is Rigshospitalet in Copenhagen, Denmark.

The logistics of patient transport and specialist care in Greenland are extremely complex, and it may take days to get severely ill or injured patients to a hospital of the appropriate size. Almost all AMSs in Greenland are coordinated by qualified personnel on duty at Dronning Ingrids Hospital. Air Greenland is the flight operator for domestic patient transports, and the aircraft most often utilized is a Beechcraft B-200, which can take 1 or 2 patients on stretchers, or 1 incubator. This aircraft is also used for passenger transport and is therefore not always available; larger Dash airplanes are used when needed. Helicopters are frequently needed to transport patients from the villages to airports for pickup by fixed-wing aircraft. The total number of stretcher patients transported in this way is close to 220 per annum. Health care staff (physician and/or nurse) from the anaesthetic department at Dronning Ingrids Hospital manage approximately 75 of those flights for the most severely ill and injured patients; on other missions, health care staffing varies. About 25 patients are intubated prior to transfer each year, and approximately 50 patients require transport to other countries for advanced care. Two thirds of these are transported to Copenhagen, a one-way distance of almost 3,500 km (Table I). These patients are usually transported first to Kangerlussuaq airport (Søndre Strømsfjord) by turboprop aircraft and onward from there by jet, either commercial Air Greenland passenger jet or a dedicated Hawker 800 air ambulance, to Copenhagen. In the best-case scenario, the transport time to Rigshospitalet is 6–7 hours, but this transfer can often take much longer. These estimates do not take account of response times, which may often include bringing the aircraft from Scandinavia to Greenland, and total transport times for medical emergencies are often counted in days.

**Table I:** Distances between some regional airports in kilometres^a^

	Greenland		Iceland		Faroe Island	Denmark
	
	Nuuk	Kangerlussuaq	Reykjavík	Akureyri	Vágar	Copenhagen^c^
Nuuk		317	1,419	1,579	2,206	3,531
Kangerlussuaq^b^	317		1,342	1,453	2,108	3,421
Reykjavík	1,419	1,342		267	789	2,113
Akureyri	1,579	1,453	267		663	1,968
Vágar	2,206	2,108	789	663		1,326
Copenhagen^c^	3,531	3,421	2,113	1,968	1,326	

a From Ref. (19).

b Søndre Strømfjord.

c Roskilde Airport.

Each year, approximately 15 patients from Greenland are brought by air ambulance from Iceland to Landspítali in Reykjavík. In recent years, patients have sometimes been transported by an Air Greenland Beechcraft B-200 from Greenland to Keflavík or Akureyri airport, where they are transferred to an Icelandic plane for onward transit to Copenhagen.

### Iceland

With 640 hospital beds and 20 surgical theatres, Landspítali in Reykjavík serves as the main tertiary hospital for the Icelandic population of 321,000 and as a secondary hospital for the 200,000 living in the capital area. This is Iceland's only university hospital, and, with 4,500 employees, it is the largest organization in Iceland. The country's second largest hospital, with 138 patient beds, is Akureyri Hospital in the north.

Landspítali is the only major hospital in the 3 countries with an almost full complement of services, including paediatrics, obstetrics, general medicine, gynaecology and advanced intensive care for adult and paediatric patients (Table II). More than medical specialties are represented, with many sub-specialties, and the hospital is capable of managing most clinical problems, sending relatively few patients abroad for treatment. Almost all specialist medical training takes place abroad, mostly in Scandinavia, the USA, the UK and the Netherlands.

**Table II:** **. Characteristics of the 3 countries and services available at the main hospitals

	Greenland	Iceland		Faroe Islands
Areal (km^2^)	2,166,086	103,000		1,399
Inhabitants (2013)	56,370	321,857		48,197
Hospital	Dronning Ingrids Hospital	Landspítali	Sjúkrahúsið á Akureyri	Landssjúkrahúsið
Beds	190	640	138	160
Blood bank	Limited services^a^	Comprehensive	Limited services^a^	Comprehensive
MRI and CT scanners	Yes	Yes^b^	Yes	Yes
Invasive radiology	No	Yes	No	No
Haemodialysis	No	Comprehensive^c^	Yes	Yes
Trauma care	Limited services	Comprehensive	Limited services	Limited services
PCI	No	Yes	No	No
Cardiothoracic surgery	No	Comprehensive^c^	No	No
Neurosurgery	Emergency	Comprehensive^c^	No	Emergency
Intensive care	Limited services	Comprehensive^c^,^d^	Limited services	Limited services
Neonatal intensive care	Limited services	Comprehensive^c^	Limited services	Limited services

CT: computed tomography; MRI: magnetic resonance imaging; PCI: percutaneous coronary intervention.

a Platelets not available.

b Not coiling of cerebral aneurysms.

c Special unit, consultants available 24/7.

d Including extracorporeal membrane oxygenation (ECMO), intra-aortic balloon pump (IABP) and continuous renal replacement therapy (CRRT).

There are plans for a new hospital building, to be launched in 2020, adjacent to Reykjavík airport. Mýflug, the company holding the Ministry of Health contract for fixed-wing AMSs, operates most of the air ambulance missions (Table III). With headquarters in Akureyri, Mýflug has one Beechcraft King Air B-200, a dedicated and well equipped air ambulance configured to transport 2 patients. Approximately 450 patients are transported per year. An emergency medical technician (EMT) follows each patients, accompanied by a physician in 40–45% of cases. An increasing number of patient transfers between hospitals in Iceland are repatriations of patients previously transferred.

**Table III:** Summary of AMSs organization in the 3 countries

	Greenland	Iceland		Faroe Islands
	
	Nuuk	Reykjavík	Akureyri	Tórshavn
Aircraft operator	Domestic (Air Greenland) From Iceland From mainland Europe	Icelandic Coast Guard Local (Eagle Air)	Local (Myflug Air and Norlandair)	Domestic (Atlantic Airways) From Iceland From mainland Europe
Personnel on call for AMS	Yes	Yes	Yes	No
Patient transport office^a^	Yes	Yes	No	Yes
Patient transports^b^				
Transatlantic	50^c^	15	0	125
Domestic – fixed wing	220	0	450	0
Domestic – helicopter	15	75	0	80
Crew configuration	Anaesthesia nurse and physician (resident or specialist) on all missions.	Emergency technician and a physician (resident or specialist doctor) on all missions.	Emergency technician on all missions, physician (resident or specialist doctor) *ad hoc*.	Normally anaesthesia nurse on domestic flights. Anaesthesia nurse and anaesthetist when needed on flights to Denmark.
National standards for crew member qualifications	No	No		No
System performance indicators		Yes^d^	Yes^d^	

a Dedicated personnel coordinating patient transport.

b Approximate number per annum, sorted by patient origin.

c Approximately 30–35 patients are transferred to Denmark and 15 to Iceland.

d Only for dispatch time.

The Coast Guard operates 3 Super Puma helicopters and rescue (SAR) helicopters and 1 Dash 8 airplane, which can take 4 patients on stretchers. There is a physician and an EMT on call for Coast Guard helicopter flights, with 2 separate call lines for AMSs in Iceland, 1 in Akureyri and 1 in Reykjavik. There are approximately 150 helicopter missions annually: half of these involve patient transport while the other half are SAR only. Other flight operators who occasionally provide airplanes for AMSs are Nordlandair (a subcontractor for Air Greenland) in Akureyri and Ernir (Eagle Air) in Reykjavík. Nordlandair operates 1 Beechcraft King Air B-200 and 3 Twin Otters. Ernir operates a Jetstream 32 for scheduled flights and, on rare occasions, as an air ambulance, mostly for transfers from Landspítali to hospitals in Scandinavia. The total annual number of patient transfers, domestic and to or from other countries, is about 550. However, most of these are secondary transports, and many are less urgent cases. No more than 5–8% of all cases are highly “time dependent” [e.g. STEMI (ST segment Elevation Myocardial Infarction), severe trauma, stroke, respiratory failure and pregnancy or childbirth].

### Faroe Islands

The Faroe Islands are located in the North Atlantic Ocean, approximately midway between Norway and Iceland. The total area of the archipelago is approximately 1,400 km^2^, with a population of almost 50,000 people. There are 19,000 inhabitants in Tórshavn and 5,000 in Klaksvík. Seventeen of the 18 islands are inhabited. The Faroe Islands are an autonomous province of the Kingdom of Denmark.

Landssjúkrahúsið in Tórshavn, with 160 beds, is the largest hospital (Table II). The 2 other hospitals are in Klaksvík (36 beds) and Suderø Sygehus in Tvøroyri (30 beds). Acute AMSs are for the most part coordinated by the patient transfers office (Uttanlandstænastan) at Landssjúkrahúsið, which has staff on call at all times. The total transport time from Tórshavn to Rigshospitalet is usually 4 hours.

The logistics of patient transport and specialist care in the Faroe Islands can also be quite complex (Table III). Atlantic Airways provides Bell 413 helicopters for both SAR and domestic patient transfers, mainly from Sudurøy (5,000 inhabitants) to Landssjúkrahúsið. The helicopters are well equipped for SAR, but carry no medical equipment other than a patient stretcher. Approximately 80 patients are transported annually by helicopter. Most are not critically ill or injured, but the alternative is a 2-hour ferry journey, which is often suboptimal. The government has agreements with 3 service providers for transporting patients abroad. A total of 2,300 patients annually travel to Denmark for health care services, and the numbers are increasing each year. Of these, about 125 are transported on a stretcher. In most cases, this is done by removing 6–12 passenger seats from one of the Atlantic Airways Airbus A319 passenger jets. In addition to the issues of patient privacy and comfort, this can be an expensive undertaking and inconvenient for other passengers if the flight is fully booked.

The government has recently reached agreements with the Icelandic Coast Guard and Norlandair in Iceland to respond to emergencies within 1 hour. However, the service contracts have not been formalized and these services are seldom utilized. Patients who are critically ill or injured are in most cases accompanied by staff from the anaesthesia department at Landssjúkrahúsið. Some cases involving newborns and children are fetched by a specialist team flying by Learjet from Copenhagen, either because of severity of illness or in cases where qualified staff at Landssjúkrahúsið are unable to accompany the patient to Rigshospitalet.

## Discussion

It is estimated that approximately 1,000 patients are transferred annually on stretchers or in incubators by AMSs from Greenland, Iceland and the Faroe Islands, including 190 transatlantic transfers. It was not possible to assess the cost of providing these services, but it is undoubtedly substantial for these small nations. A study of Scandinavian pre-hospital, physician-manned emergency medical services indicates an average of 249 annual service hours per 100,000 inhabitants (11). We believe that the number of service hours per capita is at least 2–3 times higher in our countries because of the long transport times. Among the many ways in which this important and costly part of health care provision can be improved, the present review offers 3 recommendations, all of which depend on inputs from politicians and policy makers.

First, there is a lack of legislation in relation to AMSs: there are, for example, no national standards for health personnel accompanying critically ill and injured patients, and such standards and performance measures are needed. It is the responsibility of the health care system to ensure that the appropriate personnel, equipment, training and support are available for AMSs. Reports from other countries state that, in the past, inexperienced doctors with inadequate training, supervision and equipment carried out many of the transfers (20–22). This also applies in the Arctic Region, although the situation has improved in recent years. Failure to provide patients with appropriate treatment is not acceptable.

Patient transfers often occur outside of normal working hours and take place at short notice (20). During transfer, the patient is in a noisy, difficult and potentially dangerous environment, and the transferring medical team is operating independently. The safety and well-being of the patient and crew are paramount, and unorthodox solutions are sometimes necessary. For instance, in one case a medical team was sent from Akureyri to Tasiilaq to deliver twins by caesarean section. In other cases, where a team has been sent to fetch ill or injured persons from remote places in Greenland, a large fuel tank has at times been placed inside the passenger cabin of a Twin Otter airplane. Clearly, such missions can be hazardous, and AMS resources in Iceland can also be temporarily depleted. There is a need for improved personal equipment for medical crews, life rafts are sometimes left behind, there is often no access to toilets on board, communication between service providers can be limited, and the experience, training and competencies of those involved in AMSs varies greatly. All of these deficiencies have implications for the safety of both crew and patients.

Second, improvements to clinical governance should include standardization of documentation and reporting procedures, which would facilitate quality assessment and collaborative research. The infrastructure of AMSs in these 3 countries must be strengthened, and information about costs and benefits, patient safety and outcomes must be collected systematically. One significant unknown is the proportion of critically ill or injured patients dealt with by AMSs, so-called First Hour Quintets (cardiac arrest, severe respiratory difficulties, chest pain including acute coronary syndrome, severe trauma or stroke), who must be transferred for specialist care that is unavailable at the referring hospital/health care station. A recent study of pre-hospital critical care in Scandinavia found that acute critical illness or injury occurs at a rate of 25–30 per 10,000 person years (3). It is interesting that the incidence of patient encounters with anaesthesiologist-staffed pre-hospital services varied from 5 per 10,000 inhabitants in Sweden to nearly 75 per 10,000 inhabitants in Denmark (3). It would be very useful to conduct a similar study in Greenland, Iceland and the Faroe Islands. Assuming that the incidence is comparable, the expected number of pre-hospital critical illnesses or injuries would be 1,100–1,200 per year. Other disparities in the existing emergency medical systems may also impact on outcome. It is noteworthy that all the Scandinavian countries staff rapid response cars and helicopters with specially trained physicians, usually anaesthesiologists, which is seldom the case in these 3 countries (3,11,12). It seems likely that advanced medical care and rapid transport of severely ill or injured patients to appropriate facilities improves outcomes, but more research about the organization of pre-hospital critical care services is needed (23). Other factors, not least that Greenland, Iceland and the Faroe Islands are sparsely populated, remote and face difficulties with the provision of health services, must also be taken into account. AMS patient volumes are low, and solutions that work well in Scandinavia or elsewhere may not work in this setting. Clearly, many uncertainties remain. Investing in clinical governance and collaborative research will help to answer some of these pressing questions.

Third, we believe it is important to maximize regional co-operation in the provision of AMSs and acute tertiary care. Quality of outcome tends to improve with larger patient volumes, and co-operation is one way to achieve this critical mass. However, there are important obstacles and challenges to such co-operation. A report from the Organisation for Economic Co-operation and Development (OECD) listed the most important of these, which include economic competition, long distances, lack of connectivity and strong institutional and economic links with other territories (15). We share a duty to optimize resource use in health care. Each country has a finite health care budget, and this money must be spent in the most responsible way. When appropriately resourced and operated, AMSs can save lives and reduce societal costs. It is the responsibility of AMSs to respond to emergencies and make acute secondary and tertiary hospital care accessible. The service components include a communication centre, administrative staff, appropriately trained team members, reliable equipment, and education and safety programs. Some of these components are in place, but we believe an opportunity exists for increased regional co-operation, which can lead to better patient care and reduce costs for our societies. This co-operation may include delivery of patient care as well as a systematic approach, in the form of clinical governance, maintaining and improving the quality of that care. We have a duty to overcome any barriers to such co-operation, and to use the most appropriate transport mode and route for every patient. AMSs should be an integral resource within a comprehensive health care system, and integration begins with the establishment of geographic service areas. Service areas should be based on the availability of specialty centres, and on distance of travel. In this region, Landspítali is the only tertiary hospital with comprehensive services. The new hospital is still at the planning stage, affording an opportunity to adapt the proposed facility to meet the needs of the people of Greenland and Faroe Islands. The hospital is quite close to Reykjavík airport, with regular connections to Vágar in Faroe Islands and to 5 destinations in Greenland. This is important for relatives of patients, who must in many cases leave home for extended time periods and travel long distances to be able to spend time with their loved ones.

## Conclusions

It is important to optimize resource use in health care, and to continuously assess the quality and effectiveness of established practices. Medical emergencies are time-sensitive, and there is evidence that improved outcomes depend on reducing out-of-hospital time for critically ill and injured patients, bringing more medical capabilities to selected patients and providing transport to the closest centre with appropriate services. The region discussed in this paper has sparse settlement patterns. Remoteness and climatic conditions, that are often extreme, present significant challenges.

We believe it is crucial to identify and overcome barriers to further regional co-operation in providing AMSs and advanced hospital care. Institutional and organizational issues, traditions and administrative barriers must not stand in the way of best practice. Strong leadership is crucial in bringing health care leaders to the table, formalizing co-operation, and establishing contracts and agreements. Political leaders in the area have an obligation and an opportunity to improve the lives of the region's inhabitants by formulating and implementing the initiatives necessary to achieve these advances.

## References

[1] Lockey D (2009). International EMS systems: geographical lottery and diversity but many common challenges. Resuscitation.

[2] Air Medicine: accessing the Future of Health Care (2006). http://www.vdh.virginia.gov/OEMS/Files_page/Medevac/FAREWhitePaper.pdf.

[3] Kruger AJ, Lossius HM, Mikkelsen S, Kurola J, Castren M, Skogvoll E (2013). Pre-hospital critical care by anaesthesiologist-staffed pre-hospital services in Scandinavia: a prospective population-based study. Acta Anaesthesiol Scand.

[4] Lossius HM, Soreide E, Hotvedt R, Hapnes SA, Eielsen OV, Forde OH (2002). Prehospital advanced life support provided by specially trained physicians: is there a benefit in terms of life years gained?. Acta Anaesthesiol Scand.

[5] Antman EM, Hand M, Armstrong PW, Bates ER, Green LA, Halasyamani LK (2008). 2007 focused update of the ACC/AHA 2004 Guidelines for the Management of Patients with ST-Elevation Myocardial Infarction: a report of the American College of Cardiology/American Heart Association Task Force on Practice Guidelines: developed in collaboration with the Canadian Cardiovascular Society endorsed by the American Academy of Family Physicians: 2007 Writing Group to Review New Evidence and Update the ACC/AHA 2004 Guidelines for the Management of Patients With ST-Elevation Myocardial Infarction, Writing on Behalf of the 2004 Writing Committee. Circulation.

[6] Sigmundsson TS, Gunnarsson B, Benediktsson S, Gunnarsson GT, Duason S, Thorgeirsson G (2010). [Management of patients with STEMI transported with air-ambulance to Landspitali University Hospital in Reykjavik]. Laeknabladid.

[7] Sullivent EE, Faul M, Wald MM (2011). Reduced mortality in injured adults transported by helicopter emergency medical services. Prehosp Emerg Care.

[8] Galvagno SM, Floccare DJ, Scalea TM (2012). Impact of prehospital mode of transport after severe injuries: reevaluation of results. J Trauma Acute Care Surg.

[9] D'Amore AR, Hardin CK (2005). Air force expeditionary medical support unit at the Houston floods: use of a military model in civilian disaster response. Mil Med.

[10] Santry HP, Janjua S, Chang Y, Petrovick L, Velmahos GC (2011). Interhospital transfers of acute care surgery patients: should care for nontraumatic surgical emergencies be regionalized?. World J Surg.

[11] Kruger AJ, Skogvoll E, Castren M, Kurola J, Lossius HM, ScanDoc Phase 1a Study G (2010). Scandinavian pre-hospital physician-manned Emergency Medical Services – same concept across borders?. Resuscitation.

[12] Langhelle A, Lossius HM, Silfvast T, Bjornsson HM, Lippert FK, Ersson A (2004). International EMS systems: the Nordic countries. Resuscitation.

[13] OECD (2013). Health at a glance 2013: OECD Indicators. http://dx.doi.org/10.1787/health_glance-2013-en.

[14] Bjerregaard P (2011). The Arctic health declaration. Int J Circumpolar Health.

[15] OECD (2011). OECD territorial reviews: NORA region 2011. http://dx.doi.org/10.1787/9789264097629-en.

[16] Haagensen R, Sjoborg KA, Rossing A, Ingilae H, Markengbakken L, Steen PA (2004). Long-range rescue helicopter missions in the Arctic. Prehosp Disaster Med.

[17] Zakariassen E, Uleberg O, Roislien J (2015). Helicopter emergency medical services response times in Norway: do they matter?. Air Med J.

[18] Norum J, Elsbak TM (2011). Air ambulance services in the Arctic 1999–2009: a Norwegian study. Int J Emerg Med.

[19] Bureau of transportation statistics USDoT Inter-airport distance. http://www.transtats.bts.gov/Distance.asp?.

[20] Gray A, Gill S, Airey M, Williams R (2003). Descriptive epidemiology of adult critical care transfers from the emergency department. Emerg Med J.

[21] Jameson PP, Lawler PG (2000). Transfer of critically ill patients in the Northern region. Anaesthesia.

[22] Spencer C, Watkinson P, McCluskey A (2004). Training and assessment of competency of trainees in the transfer of critically ill patients. Anaesthesia.

[23] Fevang E, Lockey D, Thompson J, Lossius HM, Torpo Research C (2011). The top five research priorities in physician-provided pre-hospital critical care: a consensus report from a European research collaboration. Scand J Trauma Resusc Emerg Med.

